# Use of near-infrared imaging using indocyanine green associates with the lower incidence of postoperative complications for intestinal and mesenteric injury

**DOI:** 10.1038/s41598-021-03361-1

**Published:** 2021-12-13

**Authors:** Keishi Yamaguchi, Takeru Abe, Kento Nakajima, Chikara Watanabe, Yusuke Kawamura, Hirokazu Suwa, Yuta Minami, Kazunori Nojiri, Hidetaka Ono, Kenichi Yoshida, Hidenobu Masui, Tomoki Doi, Ichiro Takeuchi

**Affiliations:** 1 Department of Emergency Medicine, Yokohama City University Graduate School of Medicine, 4-57 Urafunecho, Minamiku, Yokohama, Japan; 2 Department of Surgery, Yokosuka Kyosai Hospital, 1-16 Yonegahama-dori, Yokosuka, Japan

**Keywords:** Trauma, Outcomes research, Colorectal surgery, Reconstruction

## Abstract

Anastomotic leakage after intestinal resection is one of the most serious complications of surgical intervention for hollow viscus injury. Adequate vascular perfusion of the anastomotic site is essential to prevent anastomotic leakage. Near-infrared imaging using indocyanine green (NIR-ICG) is useful for the objective assessment of vascular perfusion. The aim of this study was to evaluate the association of NIR-ICG with intestinal and mesenteric injuries. This was a retrospective, single-center study of patients undergoing surgery for intestinal and mesenteric injuries. NIR-ICG was used to evaluate vascular perfusion. Postoperative complications were assessed between NIR-ICG and non-NIR-ICG groups.The use of NIR-ICG was associated with a lower incidence of Clavien-Dindo grade ≥ III complications with a statistical tendency (p = 0.076). When limited to patients that underwent intestinal resection, the use of NIR-ICG was significantly associated with a lower risk of perioperative complications (p = 0.009). The use of NIR-ICG tended to associate with the lower incidence of postoperative complications after intestinal and mesenteric trauma surgery. NIR-ICG was associated with a significantly lower risk of complications in patients undergoing intestinal resection. The NIR-ICG procedure is simple and quick and is expected to be useful for intestinal and mesenteric trauma.

## Introduction

Early surgical intervention is essential for the management of hollow viscus injury (HVI). A delay in diagnosis and treatment results in peritonitis, hemodynamic instability, and increased mortality and morbidity rates^1–4^. However, because the incidence of HVI after blunt abdominal trauma is low, surgeons have limited experience in managing these injuries^5^.

Surgical interventions for HVI are associated with several complications. Anastomotic leakage (AL) after intestinal resection, especially colocolic or colorectal anastomosis, is one of the most serious and potentially life-threatening post-operative complications which has been reported to occur in 2.5%–6.6% of colonic injuries^6,7^. Adequate vascular perfusion to the anastomotic site is important to prevent AL^8,9^. One technique commonly used to assess regional intestinal vascular perfusion is subjective clinical assessment by the surgeon performing the procedure, including evaluation of the color of the serosa and mucosa, bleeding at the bowel edge, beating of the mesenteric vessels, and bowel peristalsis. However, the accuracy of this method is limited as it is strongly influenced by the surgeon's personal experience and other external factors^10^. Further, subjective assessment of vascular perfusion may be difficult during acute mesenteric ischemia, and the accuracy of intestinal survival prediction using only clinical criteria was as low as 57.7%^11,12^.

In contrast, near-infrared (NIR) imaging using indocyanine green (ICG) (NIR-ICG) is useful for the objective assessment of vascular perfusion^13^. The use of this tool could increase the accuracy of assessment of vascular perfusion status and reduce complications compared to clinical assessment. To our knowledge, however, there have been no reports on the effectiveness of NIR-ICG to prevent complications associated with intestinal and mesenteric injuries. Thus, the aim of this study was to evaluate the association of NIR-ICG with postoperative complications after surgery for intestinal and mesenteric injury.

## Results

Among the trauma patients transported to our hospital, 37 patients with abdominal trauma who required surgery for intestinal or mesenteric injury were included in this study. The NIR-ICG group included 11 patients, and the non-NIR-ICG group included 26 patients. The baseline characteristics of both groups are presented in Table 1. As the prevalence of male trauma cases was three times higher than that of females, NIR-ICG was only used in male patients. There was no difference in the mechanism of injury or the type of trauma between the two groups, and the AAST grade was significantly higher in the NIR-ICG group (p < 0.01). Regarding vital signs on admission, heart rate was higher in the non-NIR-ICG group, but there was no difference in indicators of circulatory failure including systolic blood pressure, base excess, or lactate levels between the two groups.

**Table 1:** Characteristics of the NIR-ICG and the non NIR-ICG groups.

	NIR-ICG	Non NIR-ICG	p value
**Gender (M/F)**	11/0	17/9	0.036
**Age (year)**	60 ± 16	46 ± 19	0.031
**Vital signs**			
Heart rate (/min)	79.3	101.08	0.013
Systolic blood pressure (mmHg)	105.18	112.2	0.542
Respiratory rate (/min)	26	23.42	0.938
**Blood test**			
BS (mg)	177.9	217.8	0.710
BE (mEq/L)	− 3.19	− 2.217	0.386
Lactate (mmol/L)	2.63	2.215	0.343
**Injury penetrate/blunt**	3/8	14/12	0.169
**Mechanism of penetrating injury**			
Suicidal stab wound	3	12	
Stab wound of murder	0	2	0.772
**Mechanism of blunt injury**			
Automobile	1	6	
Motorcycle	2	2	
Bicycle	1	1	
Fall	1	1	
Other	3	2	0.542
**Characteristic of injury**			
Mesenteric laceration	6	17	
Mesenteric hematoma	10	21	
Small bowel injury	6	10	
Colon injury	1	6	0.934
**AAST grade (small bowel)**			
Grade I	0	0	
Grade II	1	9	
Grade III	3	1	
Grade IV	0	0	
Grade V	2	0	
**AAST grade (large bowel)**			
Grade I	0	0	
Grade II	1	6	
Grade III	0	0	
Grade IV	0	0	
Grade V	0	0	
**AAST grade (I–V)**	3.28	2.06	< 0.01
**Oral intake**	5.64	5.42	0.150
**Length of hospital stay**	18.4	20.1	0.909

The sites of trauma were the colon (one patient), small bowel (2 patients), colon and mesenteric hematoma or injury (5 patients), small bowel and mesenteric hematoma or injury (13 patients), mesenteric hematoma or injury (15 patients), and small bowel, colon and mesenteric injuries (one patient). Regarding AAST grading for small and large bowel injury, there were 16, 4, and 2 cases of grade 2, grade 3, and grade 5 injuries, respectively.

Table 2 summarizes the postoperative complications of the NIR-ICG group and the non-NIR-ICG group. The overall rate of Clavien-Dindo (CD) grade ≥ III complications was 24.3% (9/37) and all cases were in the non-NIR-ICG group (p = 0.036). The complications included surgical site infection (SSI) in 4 cases, paralytic ileus in 2 cases, AL in 1 case, adhesive obstruction and short bowel syndrome (SBS) in 1 case, and pancreatic fistula in 1 case. Pancreatic fistula was caused by pancreatic injury and was excluded when examining the efficacy of NIR-ICG used to assess intestinal vascular perfusion. When limited to intestinal-related complications, the incidence of CD grade ≥ III was 21.6% (8/37), and all the cases occurred in the non-NIR-ICG group (p = 0.076).

**Table 2:** Postoperative complications of the NIR-ICG group and the non-NIR-ICG group.

	NIR-ICG (cases)	Non NIR-ICG (cases)	p value
**Complication (all)**	1	10	0.119
Complication (CD ≧ III)*	0	9	0.036
SSI	0	4	
Paralytic ileus	0	2	
AL	0	1	
SBS	0	1	
Pancreatic fistula	0	1	
**Intestinal complication (CD ≧ II)**	0	6	0.151
Paralytic ileus	0	4	
AL	0	1	
SBS	0	1	
**Intestinal resection**	8	9	0.069
**Complication (CD ≧ III)**	0	6	0.009
SSI	0	3	
Paralytic ileus	0	1	
AL	0	1	
SBS	0	1	
**Intestinal complication (CD ≧ II)**	0	4	0.08
Paralytic ileus	0	2	
AL	0	1	
SBS	0	1	

*Once excluded “pancreatic fistula” which is not an intestinal complication, p-value would be 0.076 for cases limited to intestinal-related complications.

When limited to intestinal complications, the rate of CD grade ≥ II complications was 16.2% (6/37), and all cases occurred in the non-NIR-ICG group (p = 0.151). The complications included paralytic ileus in 4 cases, AL in 1 case, and adhesive obstruction and SBS in 1 case.

Seventeen patients required intestinal resection. All complications of CD grade ≥ III and intestinal complications of CD grade ≥ II only occurred in the non-NIR-ICG group (CD grade ≥ III: 35.3% (6/17), p = 0.009; CD grade ≥ II: 23.5% (4/17), p = 0.08). In the NIR-ICG group, only one patient had CD grade I paralytic ileus, but no other complications were observed (Table 3).

**Table 3:** The postoperative complications of each case.

Age	Gender	Mechanism of injury	Mesenteric hematoma/injury	Small bowel injury	Colon injury	AAST	ICG	Intestinal resection	Complication (CD grade)
82	M	Motorcycle	+	+	−	V	+	+	Ileus (I)
73	M	Fall	+	+	−	II	−	+	Ileus (IIIa)
44	M	Motorcycle	+	−	−	−	−	−	Ileus (IIIa)
11	F	Automobile	+	−	+	II	−	−	Ileus (II)
57	M	Other	+	+	−	II	−	+	Obstruction (IIIb) SBS (IVa–d)
48	M	Suicide	+	−	−	−	−	+	AL (IIIb) SSI (II)
75	M	Suicide	+	−	−	−	−	−	Pancreatic fistula (IIIa)
36	F	Suicide	+	+	+	II/II	−	+	Ileus (II) SSI (IIIa)
41	M	Stab	+	−	+	II	−	+	SSI (IIIa)
33	M	Automobile	+	−	+	II	−	+	SSI (IIIa)
38	F	Suicide	−	+	−	II	−	−	SSI (IIIa)

There were no complications due to the use of ICG, and no patients in this group experienced a delay in oral intake or prolonged hospital stay.

## Discussion

This study showed that the use of NIR-ICG tended to associate with the lower incidence of CD grade ≥ III complications (p = 0.076). No CD grade ≥ II intestinal complications were observed in the NIR-ICG group. When limited to the group that underwent intestinal resection, the use of NIR-ICG significantly associated with the lower incidence of postoperative complications (p = 0.009).

Paralytic ileus was common in the non-NIR-ICG group. It is possible that a failure to identify poor vascular perfusion through subjective evaluation in this group resulted in slowing of intestinal peristalsis and paralytic ileus. In the intestinal resection group, four cases of SSIs with CD grade ≥ II were observed. All patients were in the non-NIR-ICG group. By evaluating vascular perfusion with NIR-ICG, it may be possible to avoid unnecessary intestinal resection and reduce the risk of SSI due to contamination.

AL is one of the most serious complications after intestinal resection for HVI, with a reported incidence of 2.5%–6.6% of all colonic injuries^6,7^. In addition, emergency resection is an independent risk factor for AL (relative risk 4–6, 95% confidence interval 1.9–9.8). The presence of peritonitis is also a predictor of AL^14^. Since trauma patients who require emergency surgery are presumed to be at high risk for AL, preventive measures should be implemented. Previous studies have reported that vascular perfusion is important for reducing AL^8,9^. Compared to the less reliable subjective assessments^12^, objective confirmation of good vascular perfusion in the resected bowel stump by NIR-ICG may help prevent complications due to impaired vascular perfusion. Considering the incidence of AL reported so far, further studies are needed to evaluate the efficacy of NIR-ICG for HVI.

AL increases mortality, length of hospital stay, 30-day readmission, and postoperative infection rates^14–17^. As a result, hospital costs increased by more than $25,000 in the United States^17^ and more than €50,000 in Europe^18^ when compared to low-cost ICG. Complications, not limited to AL, doubled the length of hospital stay, increased the average total cost by $25,000, and decreases total balance and turns negative, resulting in a large loss^19^. In addition, treatment of SBS requires long-term infusions, leading to an increased risk of infection and increased cost and mortality^20^. The same is true for SSI, which has disadvantages such as prolonged hospital stay, increased costs, and prolonged use of antimicrobials^21^. We believe that the use of NIR-ICG will reduce the risk of these complications and their associated consequences.

A few studies have also investigated the use of NIR-ICG for brain injuries and burns. However, there are no reports of its use in torso trauma and to the best of our knowledge, this is the first report using NIR-ICG for intestinal and mesenteric injury. The use of NIR-ICG has made it possible to objectively evaluate intestinal vascular perfusion, which was previously evaluated using subjective evaluation of mucosal color, mesenteric vascular pulsation, and intestinal peristalsis^8^. An objective evaluation of intestinal vascular perfusion can ensure sufficient perfusion at the intestinal anastomosis and avoid unnecessary intestinal resection, which may reduce the incidence of complications caused by intestinal and mesenteric injuries, such as AL and SSI. In addition to improving patient outcomes, this could shorten hospital stays, reduce costs, and optimize the use of antimicrobials.

NIR-ICG has several advantages including the ability to perform rapid evaluation of vascular perfusion within 60 s in real-time using a simple technique, and the ability to record images allowing for retrospective evaluation.

Achieving hemostasis is the mainstay of treatment in hemodynamically unstable patients. NIR-ICG should be performed in patients with stable disease. The results of this study demonstrate the feasibility of NIR-ICG in patients with intestinal and mesenteric injury with stable hemodynamics after emergency surgery.

This study has several limitations worth noting. First, this was a retrospective, single-center study; as this was not a randomized controlled trial, the selection bias could not be ruled out and we could not confirm the effectiveness of NIR-ICG. Second, our selection of patients was based on the surgeon’s evaluation. Thus, we could not exclude the possibility of categorizing a patient that did not show any impaired perfusion parameters and thereby, be considered a negative control in patients with normal bowel perfusion following mesenteric resection. Third, the number of included patients was small and further studies with a large study population warranted to validate our results. In this study, the NIR-ICG group included only males, and so randomization for gender differences is also needed. Fourth, factors other than vascular perfusion might be the cause of intestinal complications such as AL. Future studies should consider the association of other traumatic sites, operator factors, and preoperative nutritional status on the risk of intestinal complications. Fifth, the factors affecting vascular perfusion should be considered. Although none of the patients in the present study had undergone multiple abdominal surgeries in the past, the effect of multiple previous abdominal surgeries on the fluorescence intensity of ICG cannot be denied. In addition, it might be necessary to examine whether the size of the resected mesentery affects vascular perfusion evaluation. Sixth, the delayed outflow type was not examined in this study. Thus, we could not exclude the possibility of a bias, which could be attributed to a different type of perfusion mode. Seventh, a difference in the proportion of complications among subgroups was significant but underpowered, due to a limited sample size. Finally, due to the diverse background of trauma, the protocol for using NIR-ICG has not been determined. Therefore, multi-institutional randomized studies are needed to confirm whether NIR-ICG can reduce complications such as the AL rate in intestinal mesenteric injury.

## Methods

### Study design

This was a retrospective, single-center study of patients undergoing surgery due to intestinal and mesenteric injuries from December 2006 to March 2021. This observational study protocol was reviewed and approved by the Institutional Review Board, the Ethics Committee of the Yokosuka Kyosai Hospital (No. 18-14). The Ethics Committee of the Yokosuka Kyosai Hospital also approved that; the requirement for written informed consent was waived; and the individual informed consent was opted out, due to a nature of retrospective study design, per the Personal Information Protection Law and National Research Ethics Guideline in Japan. The study was conducted in accordance with the principles of the Declaration of Helsinki.

The following patient data were collected from clinical records: age, sex, vital signs, blood test results, characteristics and mechanism of injury, severity of intestinal injury, length of hospital stay, days until oral intake and postoperative complications. The inclusion criteria were (1) cases of blunt or penetrating injuries and (2) cases diagnosed with intestinal injury or mesenteric injury before or during surgery.

### Near-infrared imaging using Indocyanine green^13^

ICG is a water-soluble compound with a molecular weight of 774.96 Da and fluorescent properties. ICG binds to serum proteins that are taken up by liver cells and excreted in bile; therefore, it is widely used clinically as a test drug for hepatic and circulatory functions. ICG contains iodine and is contraindicated in patients with iodine hypersensitivity; however, it is a relatively safe reagent with low toxicity and a short biological half-life of 3–4 min in healthy adults. The rate of allergic reaction was one per 333,000.

**Figure 1:**
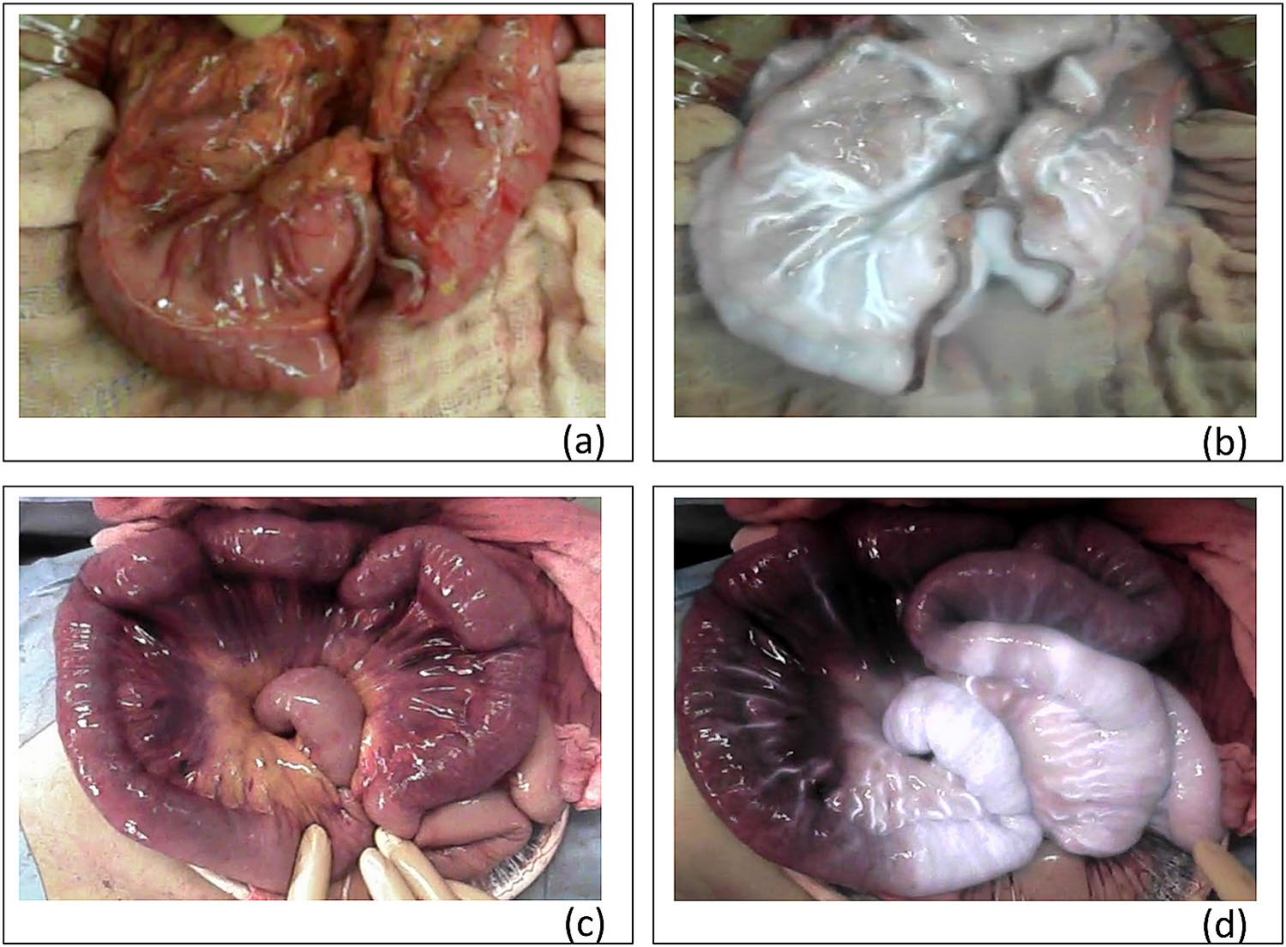
shows the assessment of vascular perfusion by near-infrared imaging using indocyanine green (NIR-ICG). After resection of the injured intestine (**a**), vascular perfusion at the stump was evaluated using NIR-ICG (**b**). Intestines that were strangled in the abdominal wall by penetrating stab wounds were evaluated (**c**), and intestines that were not well visualized within 60 s after ICG administration were resected (**d**).

The use of ICG for intraoperative perfusion angiography has been described previously^22,23^. ICG absorbs light in the NIR range of 790–805 nm, and then reemits electromagnetic energy at 835 nm. This can be visualized by fluorescence due to the NIR irradiation^13^. The HyperEye Medical System (HEMS) provided by MIZUHO Medical (Tokyo, Japan) was used at our institution. The HEMS super-sensitive optical sensor displays simultaneous fluorescence and color visible light images (Fig. 1).

### Procedure

Achieving hemostasis is the main priority in hemodynamically unstable patients. NIR-ICG should be reserved for stable patients or during planned reoperation after emergency surgery. Intestinal resection was performed in cases of grade ≥ 3 small bowel injury assessed using the American Association for the Surgery of Trauma (AAST) criteria. Colonic resection or stoma formation were performed in cases of extensive colonic injury. After resection of the injured intestine, vascular perfusion at the stump was evaluated using NIR-ICG (Fig. 1a,b). Intestinal resection was performed for mesenteric hematomas in cases of intestinal necrosis or vascular perfusion disorder. NIR-ICG was used to assess vascular perfusion where this was not clear, and in cases of intestinal strangulation by the abdominal wall due to penetrating stab wounds. If there was a fluorescence delay, the intestine was resected (Fig. 1c,d). In the assessment of vascular perfusion, no resection was performed for the normal flow type. In case of delayed inflow type, the area with impaired vascular perfusion compared to the surrounding bowel was resected. The delayed outflow type was not measured in this study^24^.

NIR-ICG was introduced into routine practice in 2013; prior to this date, the technique was used at the surgeon’s discretion. The dosage of ICG was 0.25 mg/kg. ICG dye was injected and flushed through with 20 ml of saline, following which the ICG time was started and assessed using NIR fluorescence. Time to perfusion < 60 s was considered normal, whereas a delay beyond 60 s or slower than the surrounding area suggested the presence of a vascular perfusion disorder requiring intestinal resection.

### Statistical analysis

All study variables were compared between the NIR-ICG and non-NIR-ICG groups. Statistical analyses were performed using Mann–Whitney’s *U* test for continuous variables or Fisher’s exact test for categorical variables. A detailed analysis of cases with complications was performed, using Fisher’s exact test as well. Based on observed proportions in the study sub-group, 17 patients guaranteed a power of 0.71 for 66% difference in a proportion of complications among two groups. All statistical analyses were performed using IBM SPSS Statistics for Windows, Version 25.0. (Armonk, NY: IBM Corp.). Statistical significance was set at p < 0.05.

## Conclusions

The use of NIR-ICG tended to associate with the lower postoperative complications of intestinal and mesenteric trauma. In addition, the use of NIR-ICG was associated with lower complications with statistical significance after intestinal resection. The NIR-ICG procedure is simple and quick and is expected to be useful in intestinal and mesenteric trauma. Further studies are needed to confirm whether NIR-ICG can reduce complications, such as the AL rate in intestinal and mesenteric injury.

## Abbreviations

HVI: Hollow viscus injury
AL: Anastomotic leakage
NIR: Near-infrared imaging
ICG: Indocyanine green
NIR-ICG: Near-infrared imaging using indocyanine green
AAST: American Association for the Surgery of Trauma
CD grade: Clavien–Dindo grade
SSI: Surgical site infection
SBS: Short bowel syndrome
